# Expression of purinergic receptors on microglia in the animal model of choroidal neovascularisation

**DOI:** 10.1038/s41598-021-91989-4

**Published:** 2021-06-11

**Authors:** Lu Li, Juejun Liu, Amin Xu, Peter Heiduschka, Nicole Eter, Changzheng Chen

**Affiliations:** 1 Department of Ophthalmology, Renmin Hospital of Wuhan University, 238 Jiefang Road, Wuhan, Hubei Province 430060 People’s Republic of China; 2 Department of Ophthalmology, University of Münster Medical School, Domagkstr. 15, 48149 Münster, Germany

**Keywords:** Experimental models of disease, Eye diseases

## Abstract

To investigate the effect of P2 receptor on microglia and its inhibitor PPADS on choroidal neovascularization. Forty CX3CR1^GFP/+^ mice were randomly divided into 8 groups. In addition to the normal group, the rest of groups were receiving laser treatment. The retina and choroid from the second, third, fourth and fifth group of mice were taken in the 1, 4, 7, 14 days after laser treatment. The mice in the sixth and seventh group received intravitreal injection of 2 µl PPADS or PBS respectively immediately after laser treatment. The mice in the eighth group received topical application of PPADS once per day of three days. The mice in sixth, seventh and eighth group received AF and FFA examination on the fourth day after laser treatment. Immunofluorescence histochemical staining and real-time quantitative PCR were used to evaluate P2 expression and its effect on choroidal neovascularization. After laser treatment, activated microglia can express P2 receptors (P2X4, P2X7, P2Y2 and P2Y12). The expression of P2 increased on the first day after laser damage, peaked on the fourth day (t_P2X4_ = 6.05, t_P2X7_ = 2.95, t_P2Y2_ = 3.67, t_P2Y12_ = 5.98, all P < 0.01), and then decreased. After PPADS inhibition, compared with the PBS injection group, the mRNA of P2X4, P2X7, P2Y2 and P2Y12 were decreased significantly in the PPADS injection group (t_P2X4_ = 5.54, t_P2X7_ = 9.82, t_P2Y2_ = 3.86, t_P2Y12_ = 7.91, all P < 0.01) and the PPADS topical application group (t_P2X4_ = 3.24, t_P2X7_ = 5.89, t_P2Y2_ = 6.75, t_P2Y12_ = 4.97, all P < 0.01). Compared with the PBS injection group, not only the activity of microglia cells but also the leakage of CNV decreased significantly (P < 0.01) in the PPADS injection group and the PPADS topical application group. But between two PPADS groups, the leakage of CNV had no difference (P = 0.864). After laser induced CNV, activated microglia can express P2 receptors. The P2 receptor inhibitor, PPADS, can significantly affect the function of microglia and inhibit the formation of choroidal neovascularization.

## Introduction

Choroidal neovascularisation (CNV) is the complication of many ocular fundus diseases, such as wet age-related macular degeneration (wAMD), pathologic myopia (PM), punctate inner choroidal angiopathy (PIC), choroidal rupture, etc. It represents the leading cause of irreversible blindness or low vision in developed countries^1^. Numerous researches are likely to predispose an individual to CNV, which is considered an extremely complex, multifactorial disease, but the definite pathogenesis of CNV remains unclear.

Retinal microglia are resident immune cells in the retina. Distributed in a regular array throughout the inner retina, retinal microglial cells, through their dynamic process movements, carry out constant and dynamic immune surveillance of the extracellular environment and can respond rapidly to tissue injury by altering their activation state, acquiring capabilities of migration and proliferation, as well as secreting inflammatory mediators and neurotrophic agents^2^. Found throughout the central nervous system (CNS), microglia are capable of carrying out diverse sets of housekeeping functions under normal conditions and also executing adaptive functions under conditions of tissue injury^3^. A lot of studies reported that microglial cells play important roles in the physiological process of aging retina and pathological process of retina from wAMD patients and animal models^4,5^.

Many questions still remain that what the exact function of microglia in pathogenesis of CNV and how it changes its behaviour in the diseased retina compared to a healthy one. Purinergic (P2) receptor, one of receptors on the microglial cells, has already been confirmed to be of critical importance for microglial cells in the CNS^6–8^. P2 receptors also exist on the microglial cells in the retina. However, some further investigations are still needed to confirm whether the inhibition of P2 receptors can influence the behaviour of microglial cells or change the pathological situation of CNV formation. In this study, we used the established experimental model of laser-induced CNV in mice. After treatment with a laser, CNV and tissue proliferation occur in the laser spots. It has been shown that microglia were accumulating in the laser spots^9^. For the activated microglial cells in the laser spot, we studied whether there were P2 receptors expressed by microglial cells, and whether PPADS (pyridoxalphosphate-6-azopheny 1-2’,4’-disulfonic acid), a P2 antagonist, could influence the behaviour of microglia and have effect on the CNV.

## Materials and methods

### Animals

40 six to eight weeks old CX3CR1^GFP/+^ mice were used in this experiment, and all mice were bred and kept under IVC conditions. The CX3CR1^GFP/+^ mice were supplied by Prof. Heiduschka who was also the fourth author in this manuscript. The mice were divided into 8 groups randomly. There were five mice in each group, which included two or three female mice. This research followed the local animal ethics procedures and was approved by the ethics committee of Renmin Hospital of Wuhan University and University of Münster Medical School. All experiments were performed in accordance with the ARVO Statement for the Use of Animals in Ophthalmic and Vision Research and the EU directive 2010/63/EU. This study was carried out in compliance with the ARRIVE guidelines.

### Mouse model of laser-induced CNV

Except for the normal group without any treatment (5 mice), the mice in the other 7 groups were receiving laser treatment. Mice were firstly anaesthetised by an intraperitoneal injection of a mixture of 130 mg/kg ketamine and 2.7 mg/kg xylazine. The pupils of mice were fully dilated with 1% tropicamide eye drops and 5% neosynephrine eye drops. Before placed in front of the slit lamp for laser treatment, the cornea of mice was anaesthetised with 0.5% proparacaine eye drops. Both eyes of mouse were treated with a 532 nm argon green laser (NIDEK MC-500 Vixi, Nidek Co., Ltd. Gamagori, Japan) through dilated pupils with a coverslip over the cornea to create breaks in Bruch’s membrane with a central bubble formation. The laser spots were placed on the retina around the optic nerve head between the large vessels. There were 7 laser spots in each eye. The laser beam had a diameter of 75 µm, duration of 100 ms and energy of 200 mW.

### Grouping and intervention

The first group is the normal group without any treatment. The specific treatment approach of each group can be referred to Table 1. After treated with a laser to induce CNV, animals in the second, third, fourth and fifth group received no subsequent treatment, and eyes were isolated at 1, 4, 7 and 14 days after laser treatment. Mice in the sixth, seventh and eighth group received intravitreal injection of 2 µl PPADS (sc-202770A, Santa Cruz Biotechnology) (PPADS injection group) or phosphate buffered saline (PBS) (PBS injection group) immediately after laser treatment or eye drops of PPADS (PPADS topical application group) once per day after laser treatment until the third day, respectively. For intravitreal injections, a 1 mm aperture was made posterior to the superotemporal limbus with a sharp 30-gauge needle (Hamilton, Switzerland). Then a blunt 32-gauge needle (Hamilton, Switzerland) was inserted through this opening, and PPADS solution or PBS was injected slowly. The needle was left inside the eye for additional 3–4 s to minimise reflux and to allow for diffusion of the liquid. At 4 days after laser treatment, mice in PPADS injection group, PBS injection group and PPADS topical application group received scanning laser ophthalmoscopy examination. Then eyes were isolated to prepare cryosections for immunohistochemistry and PCR. For each mouse in all groups, one isolated eye was randomly selected for immunohistochemical staining, and the other eye for PCR detection.

**Table 1:** The specific treatment approach of each group.

Groups	1st group	2nd group	3rd group	4th group	5th group	6th group	7th group	8th group
	(Normal group)	(Day 1 group)	(Day 4 group)	(Day 7 group)	(Day 14 group)	(PPADS inj. group)	(PBS inj. group)	(PPADS topical application group)
Laser	No	Laser	Laser	Laser	Laser	Laser	Laser	Laser
Treatment	No	No	No	No	No	PPADS inj	PBS inj	PPADS eye drops
Day 1	TS	TS						PPADS eye drops
Day 2								PPADS eye drops
Day 3								PPADS eye drops
Day 4			TS			TS	TS	TS
Day 7				TS				
Day 14					TS			

*TS* take samples.

### Scanning laser ophthalmoscopy

Before examinations, mice were anaesthetised and pupils were dilated by the method described before. Green fluorescent protein (GFP) fluorescence and fluorescein angiography (FA) were performed with Heidelberg Retina Angiograph II from Heidelberg Engineering (Heidelberg, Germany). Fluorescein sodium (2 mg/kg, Pfaltz & Bauer, Waterbury, CT, USA) were delivered into mice by intraperitoneal injection. Immediately after injection, infrared reflectance recordings of the mouse retina were detected with a diode laser source of 815 nm wavelength. For GFP fluorescence imaging, an optically pumped solid state laser (OPSL) source was used to generate a blue light excitation wavelength of 488 nm. Emitted light was detected between 500 and 700 nm with a detection efficiency of 85% for the fluorescence images. In FA images, the presence of an increasing hyperfluorescent lesion at the site of laser spot was defined as leakage from CNV. The whole procedure of examination took approximate eight minutes after intraperitoneal injection of fluorescein. In order to improve the signal-to-noise ratio of the fluorescence signal, a mean image was calculated from several images using suitable image-analysis software (Heidelberg Eye Explorer, Heidelberg Engineering).

### Eye cryosections

Mice were killed by cervical dislocation. Eyes were isolated, embedded in Tissue-Tek and immediately frozen in liquid nitrogen. Sections of 10 µm thickness were made using a cryostat, mounted on glass slides and stored at − 20 °C until further use.

### Immunohistochemistry

Before staining, cryosection slides were dipped in − 20 °C cold methanol for 10 min for fixation and antigen retrieval. After rinsing with PBS, sections were blocked using Power Block™ reagent (HK085-5K, BioGenex) for 6 min and incubated overnight at 4 °C with primary antibodies. The sections were washed three times with 0.1 M PBS and then incubated with two different appropriate secondary antibodies at 1:200 dilution for 1 h at room temperature at the same time. The nuclei were counterstained with DAPI (*4*′*6*′-diamidino-*2*-phenylondole dihydrochloride) for 8 min at room temperature. Finally, sections were washed and mounted under glass coverslip using mounting media. Both primary antibodies and secondary antibodies were diluted with 1% bovine serum albumin (BSA). A summary of all antibodies used was listed in Table 2. Negative controls were obtained by staining procedures that omitted the primary antibody. A fluorescence microscope (EVOS fl, Advanced Microscopy Group, USA) was used to acquire images.

**Table 2:** Primary and secondary antibodies used in this study.

Antibody	host	dilution	source	No
**Marker for microglia**				
Iba1	rabbit	1:500	Wako	019-19741
**Purinergic receptors**				
P2X4	goat	1:10	Santa Cruz Biotechnology	sc-15187
P2X7	goat	1:10	Santa Cruz Biotechnology	sc-15200
P2Y2	rabbit	1:50	Santa Cruz Biotechnology	sc-20124
P2Y12	goat	1:10	Santa Cruz Biotechnology	sc-27152
**Secondary antibodies**				
Anti rabbit Cy3	goat	1:200	Life technologies	A10520
Anti goat AlexaFluor 488	donkey	1:200	Life technologies	A11055
Anti rabbit AF 488	donkey	1:200	Jackson ImmunoResearch	712,547,003

### Reverse transcription and real-time quantitative PCR

Total RNA was extracted from retina and choroid of eyes in eight groups using TRIzol reagent (Invitrogen) following the manufacturer’s instructions. 2 μg total RNA from each sample was reverse-transcribed to cDNA with Easy-Script First-Strand cDNA Synthesis SuperMix (TransGen Biotech). The gene-specific primers (SBS Genetech) were listed in Table 3. RT-PCR was performed using SYBR Green RT-PCR Master Mix (TransGen Biotech) according to the manufacturer’s instruction. The GAPDH was set as the internal control gene in the animal and cellular experiments. The relative quantity of mRNA expression was calculated according to the formula: 2^-(targetgeneCt – GAPDHCt)^ * 10^3^, in which Ct was the threshold cycle number. All assays were repeated at least in triplicate independently. The RNA from different animals was measured individually.

**Table 3:** Primer sequences for PCR analysis.

Target	Primer sequence (5’- 3’)	Length (bp)
P2X4	Forward TGTCCCCAGGCTACAATTTC Reverse GGCAGCTTTTTCTCCCTTCT	373
P2X7	Forward CTTTTGCACCTTGAGCTTCC Reverse TCCATGCTAAGGGATTCTGG	152
P2Y2	Forward GTGGCCTACAGCTTGGTCAT Reverse GCGTGCGGAAGGAGTAGTAG	235
P2Y12	Forward AGTGATGCCAAACTGGGAAC Reverse TGAATGCCCAGATAACCACA	208

### Data analysis

Adobe Photoshop CS5 (Adobe, San Jose, CA) was used to quantify the leakage of fluorescence from CNV and the fluorescence of microglia in the laser spot by analysing the pixel intensity. Fluorescence ratio was calculated by comparing the pixel intensity of CNV or microglia in the laser spot with the pixel intensity of normal retina which was next to the laser spot. The statistical analysis was performed with ANOVA among PPADS intravitreal injection group, PPADS topical application group and PBS intravitreal injection group using SPSS 19.0 software package (SPSS Inc., Chicago, IL, USA). A P value of < 0.05 was considered statistically significant.

## Results

### Expression of purinergic receptors by microglia after laser treatment

**Figure 1:**
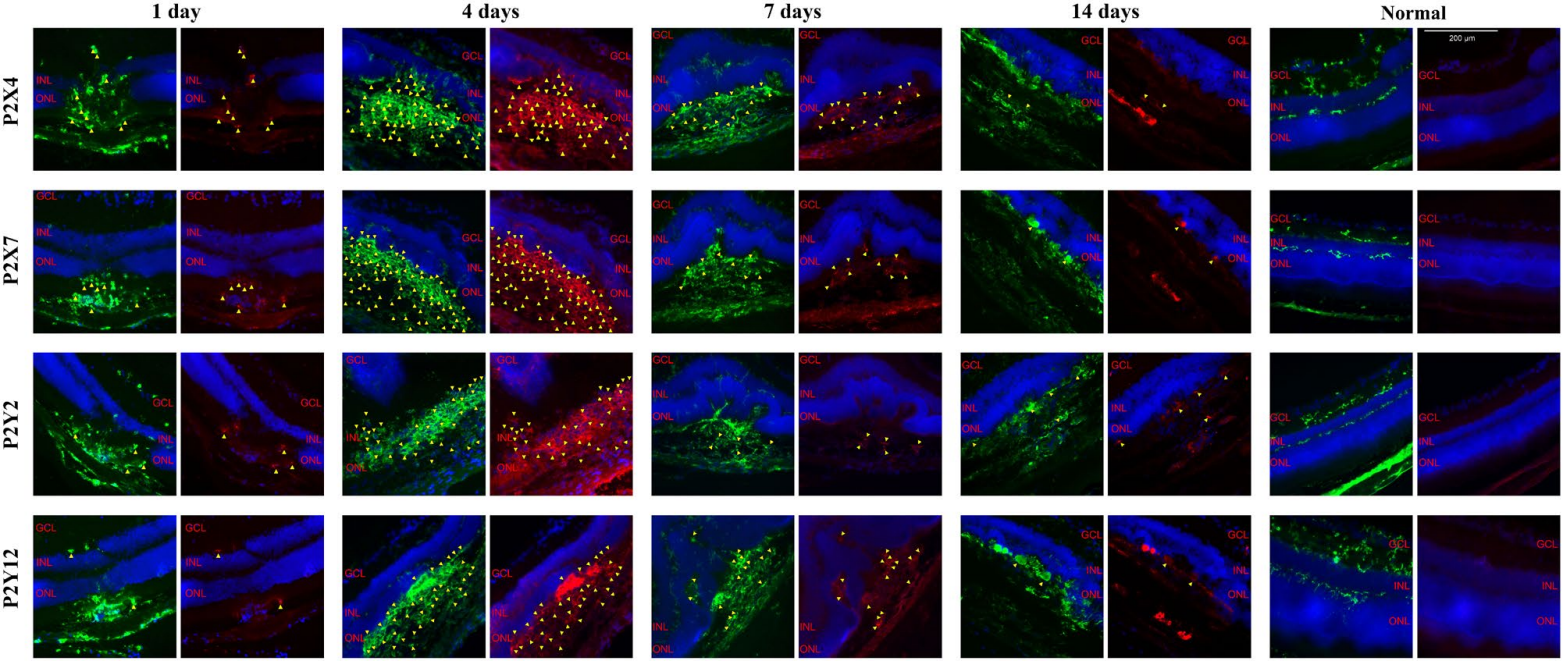
Cryosections of treated mouse eyes stained against Iba1 (green) and P2 receptors (red) on different time points after laser treatment as indicated. The arrow showed the cells which were positive for Iba1 and P2 at the same time. Scale bar: 200 µm.

The results of immunohistochemical double staining against Iba1 and purinergic receptors P2X4, P2X7, P2Y2 and P2Y12 at different time points after laser treatment were shown in Fig. 1. It was found that in the normal mouse retina, microglial cells showed almost no immunoreactivity for P2 receptors. After laser treatment, immunoreactivity for P2 receptors increased clearly, with the maximal response at day 4 after laser treatment. At day 14 after laser treatment, only several microglial cells showed immunoreactivity for P2 receptors.

**Figure 2:**
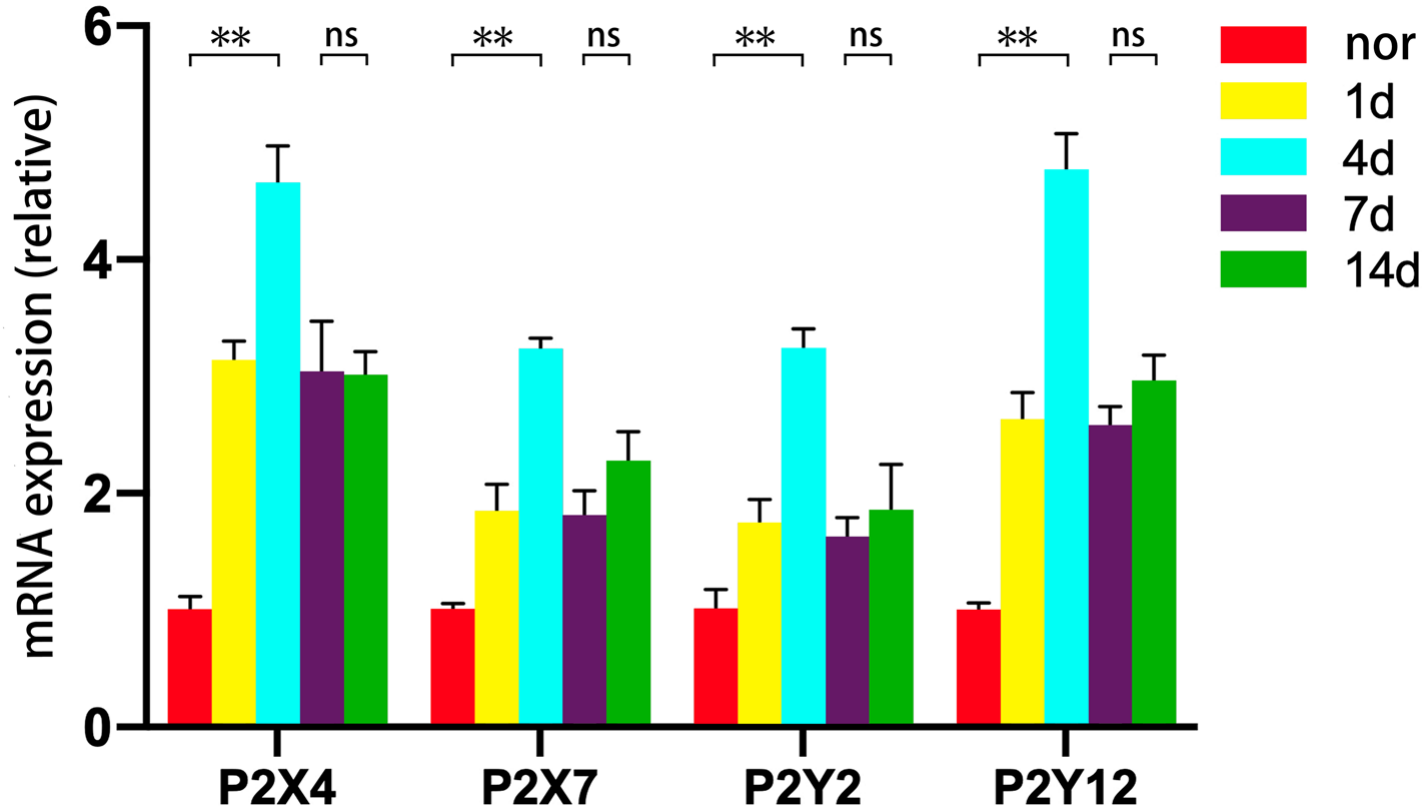
The expression level of P2 receptors’ mRNA on different time points after laser treatment. (**P < 0.01, ns (no significance) P > 0.05).

The mRNA changing levels of P2 receptors were shown in Fig. 2. After laser treatment, mRNA of P2 receptors increased clearly at day 1, with the maximal response at day 4 (t_P2X4_ = 6.05, t_P2X7_ = 2.95, t_P2Y2_ = 3.67, t_P2Y12_ = 5.98, all P < 0.01, compared with the normal group), then declined. The expression levels between day 7 and day 14 had no significantly difference (t_P2X4_ = 9.59, P = 0.057; t_P2X7_ = 11.32, P = 0.061; t_P2Y2_ = 9.41, P = 0.064; t_P2Y12_ = 6.97, P = 0.072).

### The inhibition by PPADS on purinergic receptors and microglia

**Figure 3:**
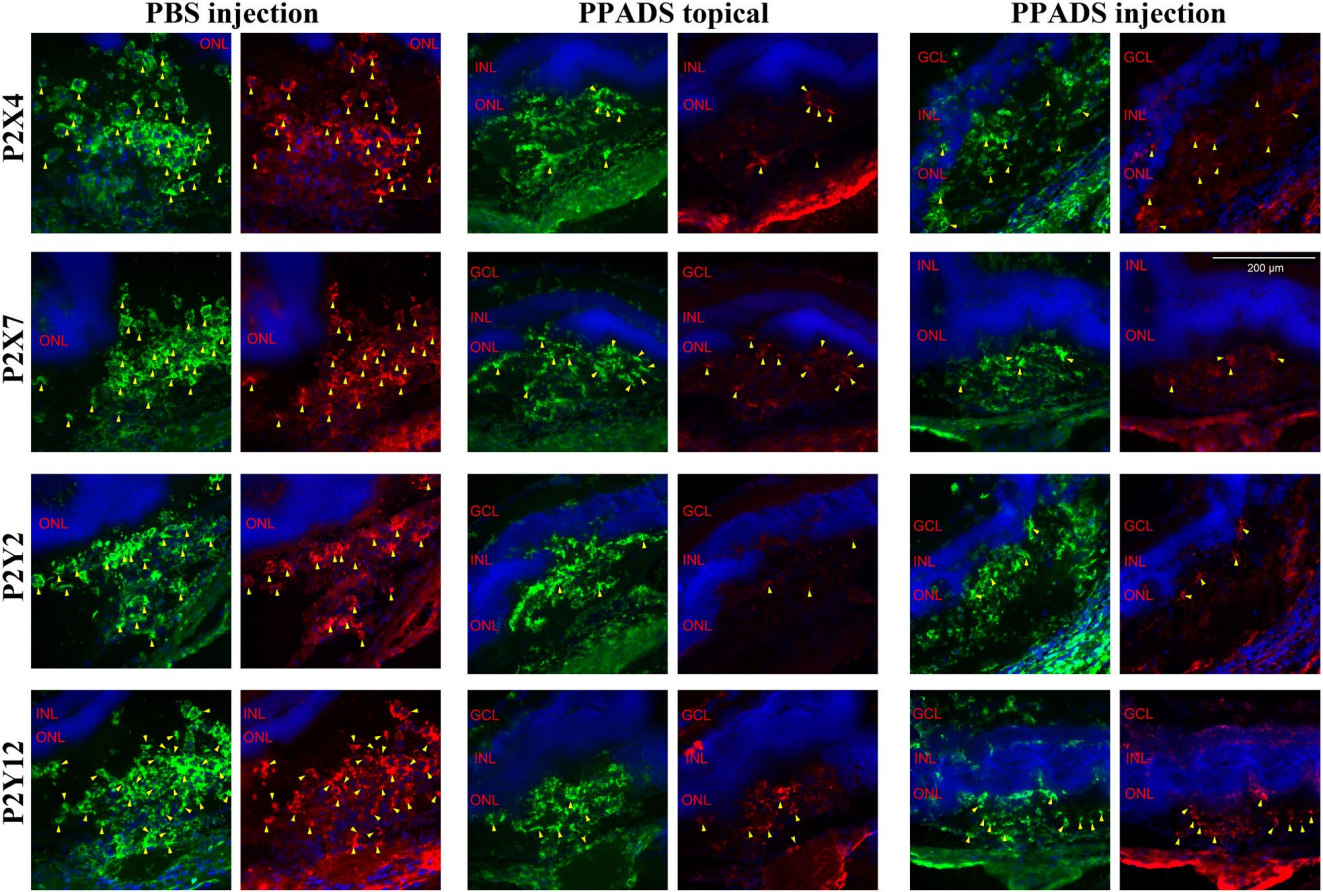
Double staining against Iba1 (green) and P2 receptors (red) of cryosections obtained day 4 after laser and drug treatment as indicated. The arrow showed the cells which were positive for Iba1 and P2 at the same time. Scale bar: 200 µm.

The results of immunohistochemical double staining against Iba1 and P2 receptors after inhibition by PPADS showed that there were less microglial cells positive for these four P2 recepors than PBS injection group, but the difference between PPADS topical application group and PPADS injection group was not very obvious. On staining pictures (Fig. 3), It was found that after treatment with PPADS, immunoreactivity for P2X4, P2X7 and P2Y2 was decreased clearly.

**Figure 4:**
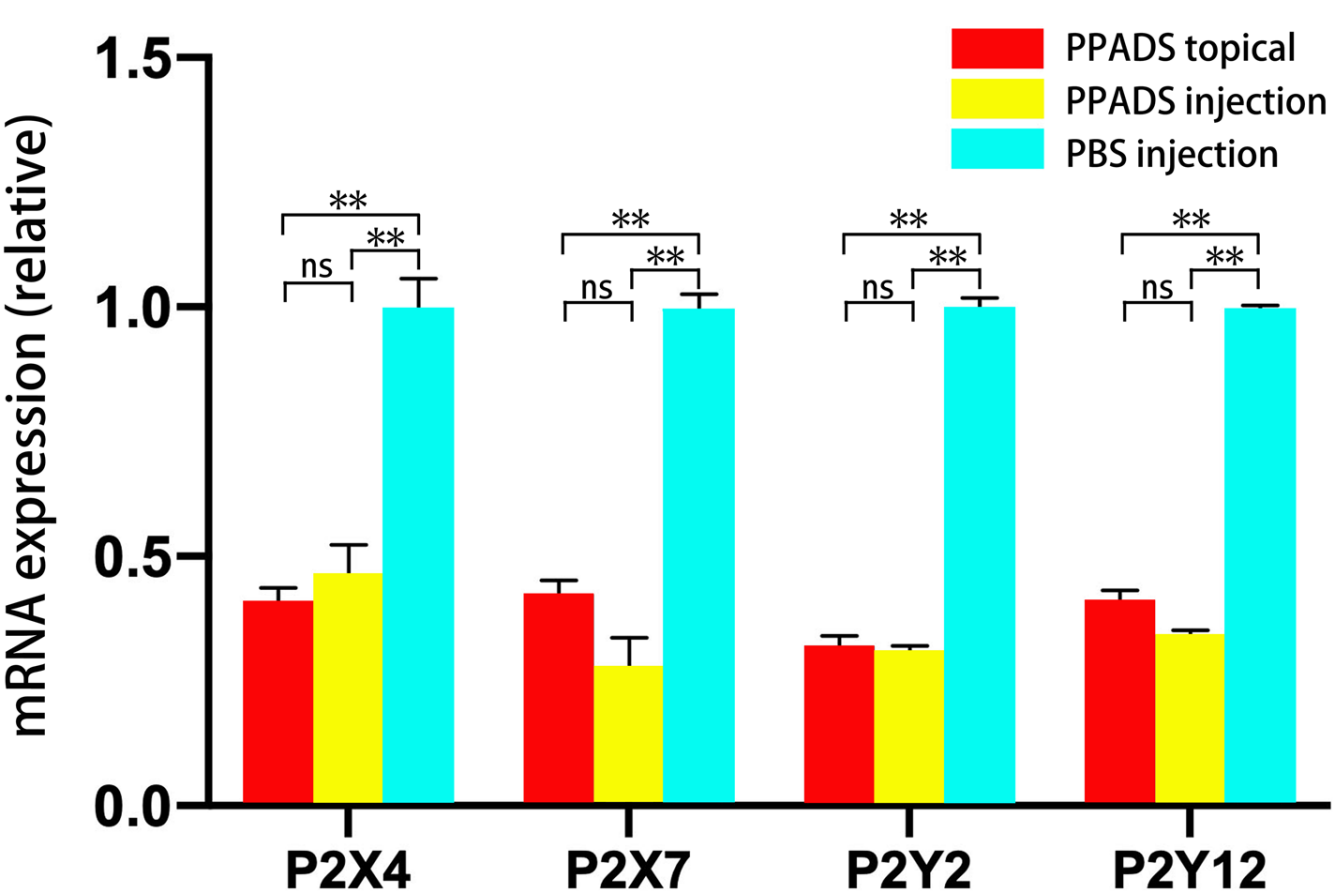
The expression level of P2 receptors’ mRNA on Day 4 after laser and drug treatment. (**P < 0.01, ns P > 0.05).

After PPADS inhibition, the mRNA changing levels of P2 receptors were shown in Fig. 4. Compared with PBS injection group, mRNA of P2 receptors decreased significantly both in PPADS topical application group (t_P2X4_ = 3.24, t_P2X7_ = 5.89, t_P2Y2_ = 6.75, t_P2Y12_ = 4.97, all P < 0.01) and PPADS injection group (t_P2X4_ = 5.54, t_P2X7_ = 9.82, t_P2Y2_ = 3.86, t_P2Y12_ = 7.91, all P < 0.01). Between different PPADS inhibition application groups, they had no differences (t_P2X4_ = 6.82, P = 0.876; t_P2X7_ = 12.32, P = 0.793; t_P2Y2_ = 10.13, P = 0.574; t_P2Y12_ = 8.79, P = 0.497).

### The leakage of fluorescence from CNV and the fluorescence of microglia after inhibition by PPADS

**Figure 5:**
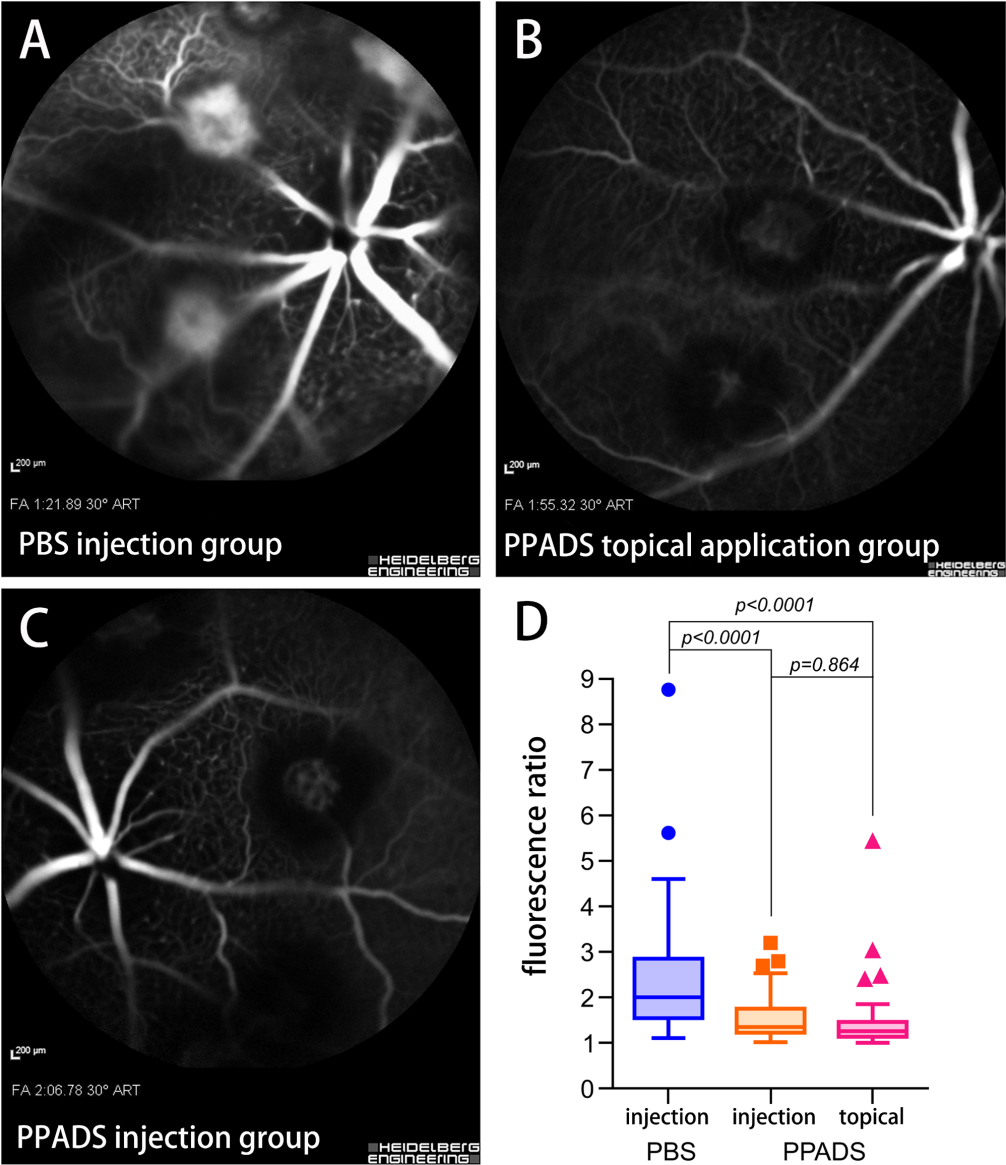
Representative fluorescein angiographs from the three experimental groups (**A** PBS injection group; **B** PPADS topical application group; **C** PPADS injection group) taken with the scanning laser ophthalmoscope. Diagram (D) shows results of numerical evaluation of intensity of fluorescence in the laser spots showing size of leakage.

The mean fluorescence ratio of CNV in the PBS injection group was 2.38 ± 1.35, 1.55 ± 0.54 in the PPADS injection group, and 1.45 ± 0.72 in the PPADS topical application group. The ANOVA of the fluorescence ratio of CNV in these three groups was 12.53 (P < 0.001). The mean fluorescence ratio of CNV in PPADS topical application group (P < 0.001) and PPADS injection group (P < 0.001) were significantly decreased compared with the mean fluorescence ratio of CNV in PBS injection group. The mean fluorescence ratio of CNV had no significantly difference between two PPADS inhibition groups (P = 0.864) (Fig. 5).

**Figure 6:**
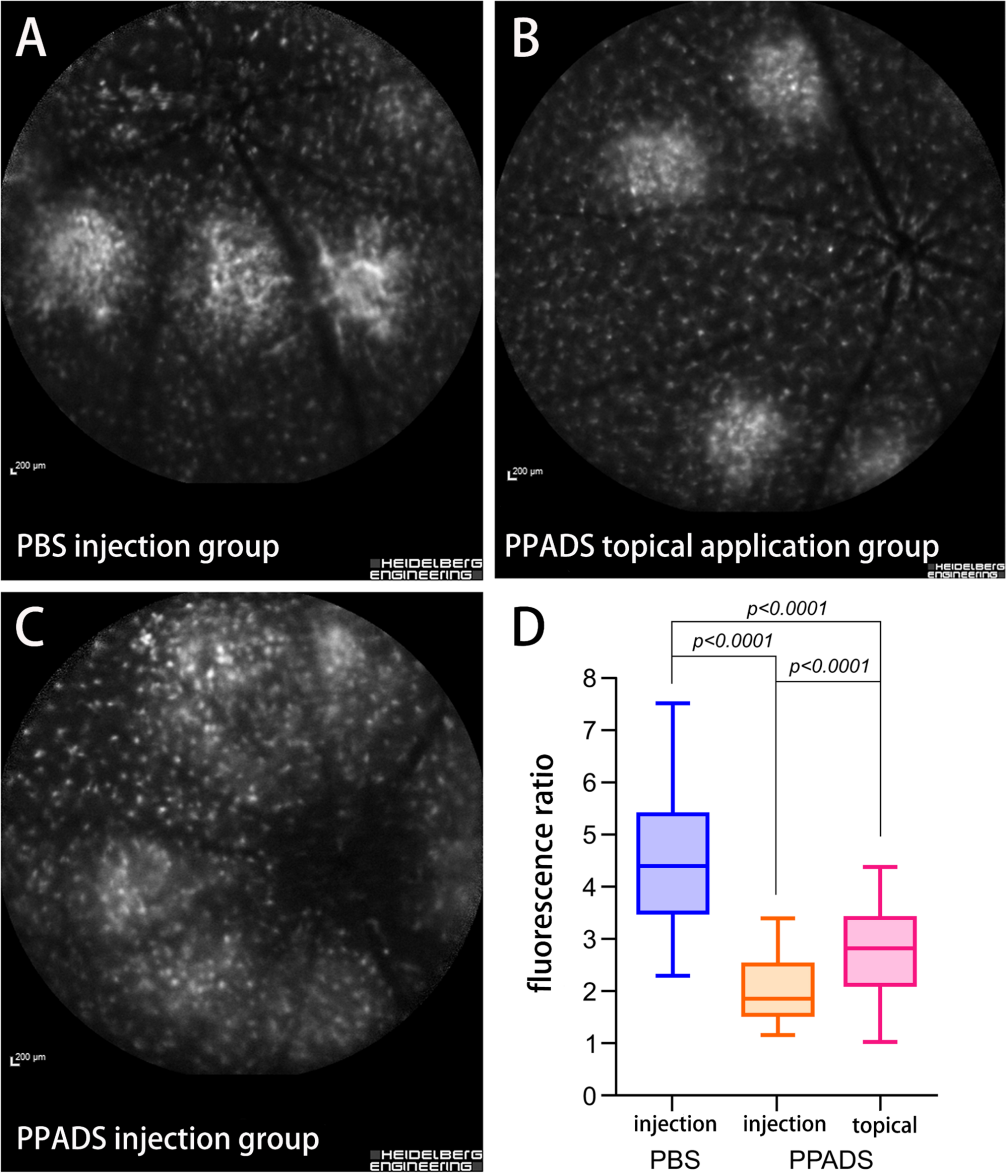
Representative images of GFP fluorescence of microglia in the laser spots in three experimental groups (**A** PBS injection group; **B** PPADS topical application group; **C** PPADS injection group) taken with the scanning laser ophthalmoscope. Diagram (D) shows results of numerical evaluation of intensity of fluorescence in the laser spots showing amounts of microglial cells that migrated into the laser spots.

The mean fluorescence ratio of microglial cells in the PBS injection group was 4.54 ± 1.22, 1.99 ± 0.62 in the PPADS injection group, and 2.74 ± 0.84 in the PPADS topical application group. The ANOVA of the fluorescence ratio of microglial cells in these three groups was 101.151 (P < 0.001). The mean fluorescence ratio of microglial cells in PPADS topical application group (P < 0.001) and PPADS injection group (P < 0.001) were significantly decreased compared with the mean fluorescence ratio of microglial cells in PBS injection group. (Fig. 6).

## Discussion

Purinergic receptors are a family of plasma membrane molecules in almost all mammalian tissues^10^. According to different activation, purinergic receptors included P1 receptors which response to the release of adenosine and P2 receptors which response to the release of adenosine triphosphate (ATP)^10^. P2 receptors have two different subtypes, P2X and P2Y. P2X receptors (P2X1-P2X7) are coupled to non-selective cation channels, and P2Y receptors (P2Y1, P2Y2, P2Y4, P2Y6, P2Y11, P2Y12, P2Y13 and P2Y14) are G protein-coupled^11^. Inoue examined the microglia in a primary culture from rat brain and found that the microglia in the rat brain expresses mainly mRNAs of P2X4, P2X7, P2Y2, P2Y6 and P2Y12^11^. In our study, we chose P2X4, P2X7, P2Y2 and P2Y12 as the main research targets, and found that these four purinergic receptors were also expressed on mouse retinal microglial cells.

P2X4 receptor has been confirmed to have the physiological functions of modulation of neurotransmission and synaptic strengthening in the CNS^12^. In some studies, it is also reported that P2X4 receptors influence the inflammasome signalling after CNS injury^13^. Ho et al*.* studied the distribution of P2X4 receptors in the mouse retina using fluorescence immunohistochemistry and demonstrated that the staining of P2X4 receptors was co-localised with microglial cells^14^. Volonté et al*.* summarised the characteristic of P2X7 receptor and demonstrated that P2X7 can regulate the immune function and inflammatory responses as well as affect neuronal cell death in CNS and other systems^15^. Vessey and Fletcher performed immunohistochemical staining and concluded that in the mouse retina, microglial cells can express P2X7 receptors^16^. Gu et al*.* studied the relationship between P2X and AMD in human blood and primate eyes and found that in AMD patients, the P2X4 Tyr315Cys variant was twofold more frequent than in age-matched control subjects, and the P2X7 Gly150Arg was also overrepresented in patients with AMD^17^. In primate eyes, both P2X4 and P2X7 receptors were detected on microglial cells immunohistochemically^17^. When these two receptors were unactivated, they can act as scavenger receptors in the eye to clear the debris and removal of subretinal deposits. Once activated, they may cause the loss of innate phagocytosis and predispose individuals towards AMD^17^. This conclusion was similar with our study that the expression of P2X4 and P2X7 was significantly high in laser-induced CNV model than in normal mice, which was a typical sign of wAMD.

P2Y2 and P2Y12 were reported to affect the activation and chemotaxis of microglia in brain^18^. Kobayashi et al*.* investigated the partial sciatic nerve ligation model in the rat and found that in the spinal cord, P2Y12 mRNA and protein increased dramatically and peaked at day 4 after injured^19^. They also confirmed that the cells expressing increased P2Y12 after nerve injury were exclusively microglia^19^. The response time and the source of P2Y12 in our research were in accordance with this finding. However, about the expression of P2Y2 and P2Y12 receptors on microglial cells in the retina in physiological or pathological conditions, limited information can be consulted. In the laser-induced CNV mouse model we applied in our study, the expression of P2Y2 and P2Y12 on microglial cells increased and reached the peak at day 4 after laser treatment. At different time points we checked, most of P2Y2 and P2Y12 receptors were expressed by microglial cells, especially at day 1 and day 4 after treatment.

We confirmed the expression of P2X and P2Y receptors on microglial cells in the animal model of laser-induced CNV. In our study, in the normal mouse retina, there were no microglial cells express P2X4, P2X7, P2Y2 and P2Y12 receptors. After laser treatment, microglial cells express these four purinergic receptors, especially at day 4 after treatment. The decrease of the expression at day 7 and particularly at day 14 after laser treatment can possibly be attributed to a gradual decline of activation of microglial cells after their initial activation due to the damage elicited by the laser treatment^20^.

PPADS is an antagonist of purinergic receptors^21^. Sarman et al. found that PPADS can strongly inhibite the oxygen-induced preretinal neovascularization, and this was accompanied by a down-regulation of P2X2 receptor expression in the inner plexiform layer of mice^22^. Birke et al*.* studied laser-induced CNV mice models and performed topical application of PPADS on mice eyes for 3 consecutive days^23^. They found that PPADS can attenuate the area of CNV as well as the migration of endothelial cells, and attributed this result to the inhibition by PPADS on the complement activation and the membrane attack complex (MAC) formation^23^. In our research, we found the inhibition by PPADS not only on the reduced leakage of fluorescence from CNV, but also on the migration of microglial cells, demonstrated by reduced fluorescence in the area of the laser spot. We speculated that the inhibition by PPADS on the CNV may partly because that PPADS inhibited the P2 receptors on microglial cells, therefore inhibited the function of microglial cells which play critical roles in the pathological process of CNV^4,20^. Two different application ways of PPADS had no significant difference in inhibition of the leakage of fluorescence from CNV, but significant difference in the inhibition of the migration of microglia, which indicated that effective inhibition concentrations can also be achieved by eye drops. This offers the possibility of non-invasive treatment in the clinic study. However, in this study, we did not evaluate the potential differences of inhibited effect between topical application and intravitreal injection. Further study on the drug permeability and mechanism of action of PPADS may should be set in order to find the equivalent inhibition level. In addition, we preliminarily confirmed the inhibited effect of PPADS on CNV and microglia cells by giving PPADS intervention immediately after laser-induced CNV formation, but the differences of inhibition in different stages of CNV formation were not compared in this study. Based on this thinking, we are considering to study the inhibited effect of P2 receptors at more time points in order to obtain more valuable information to guide our further study of this project.

## Conclusion

In our study, the microglial cells can express purinergic receptors P2X4, P2X7, P2Y2 and P2Y12 in the laser spots of mouse retina, especially at day 4 after treatment. The purinergic receptor inhibitor PPADS can significantly inhibit the formation of CNV and the migration of microglial cells both in topical application and intravitreal injection ways. Further research is needed to figure signalling pathways out in more details. Whether PPADS or other purinergic inhibitors can be used as a new therapy for CNV, it still needs more research to investigate its safety and effectiveness.

## Data Availability

The data used to support the findings of this study are available from the corresponding author upon request.
